# Association Between Vitamin D Deficiency and Sepsis in Term Neonates: A Case-Control Study

**DOI:** 10.7759/cureus.72468

**Published:** 2024-10-27

**Authors:** Jasleen Dua, Renuka S Jadhav, Mridu Bahal, Shailaja Mane, Shivani Kale, Srinija Garlapati, Md Ilyaz, Kasireddy Sravanthi, Gaurav Kumar, Ruhi Shaligram

**Affiliations:** 1 Pediatrics, Dr. D. Y. Patil Medical College, Hospital & Research Centre, Dr. D. Y. Patil Vidyapeeth (Deemed to be University), Pune, IND

**Keywords:** immunomodulatory properties, neonatal sepsis, serum levels, term neonates, vitamin d

## Abstract

Background

Neonatal sepsis remains a major global health challenge, contributing significantly to morbidity and mortality in term neonates. Despite advancements in neonatal care, the early identification and prevention of sepsis continue to pose challenges. Emerging research suggests that vitamin D, traditionally recognized for its role in bone health, also plays a crucial role in immune regulation.

Objectives

To evaluate and compare serum vitamin D levels in term neonates with sepsis and without sepsis.

Methods

The study included 60-term neonates, with 30 neonates diagnosed with sepsis as cases and 30 healthy-term neonates as controls. Detailed history and clinical examination were performed for all subjects. Sepsis was diagnosed based on clinical and laboratory criteria. Serum vitamin D levels were assessed using the chemiluminescent microparticle immunoassay (CMIA) technique.

Results

The mean serum vitamin D levels in the sepsis group were significantly lower (16.0 ng/mL ± 10.5) compared to the control group (29.07 ng/mL ± 8.4) with a p-value <0.01. There was no significant difference in gender distribution (p=0.79), socioeconomic status (p=0.752), or maternal age (p=0.349) between the groups. Significant differences were found in the mode of delivery (p=0.037), presence of meconium-stained liquor (p=0.001), intrapartum antibiotic administration (p=0.0006), and resuscitation requirements (p=0.0004). APGAR scores at one minute were significantly lower in the sepsis group (p=0.001). Clinical features among sepsis cases included poor activity (40%), tachypnoea (46.6%), tachycardia (43.3%), and hyperthermia (40%).

Conclusion

A strong association between vitamin D deficiency and the severity of sepsis was observed, with deficient neonates showing higher rates of severe, early-onset sepsis.

## Introduction

Neonatal sepsis is a clinical syndrome marked by bacteremia and systemic infection symptoms, such as septicemia, meningitis, pneumonia, and septic arthritis, occurring within the first 28 days of life. This condition leads to life-threatening organ dysfunction and is a significant cause of morbidity and mortality in infants. Neonatal sepsis is categorized into early-onset sepsis (EOS) occurring within the first 72 hours of life and late-onset (LOS), which occurs after 72 hours [1]. A global study on the incidence and mortality of neonatal sepsis reported 2,824 cases per 100,000 live births between 1979 and May 2019, with an estimated mortality rate of 17.6% [2]. In late preterm newborns, the incidence ranges from 6% to 10%, with mortality rates increasing with age, reaching 36% for newborns aged 8-14 days and 52% for those aged 15-28 days [3].

Traditionally, vitamin D has been recognized for its role in bone health and calcium regulation. However, recent research has shown that this steroid hormone plays a crucial role in the functioning of many organ systems. Vitamin D receptors (VDR) and the enzyme 25-hydroxy vitamin D-1α-hydroxylase have been found in various tissues beyond the skeleton. Additionally, the vitamin D response element (VDRE) has been identified in over 900 genes, and emerging studies suggest that adequate vitamin D levels may protect against several chronic diseases, including systemic infections, cardiovascular and lung diseases, and diabetes [4].

Vitamin D deficiency is common in critically ill children, with an incidence of 86.4% among those with sepsis. Low levels of 25-hydroxy vitamin D impair immune function, disrupt hormone metabolism, and increase the likelihood of infections and critical illnesses, ultimately contributing to higher mortality rates. Current guidelines suggest that vitamin D levels below 20 ng/mL are insufficient, while levels above 20 ng/mL are considered sufficient [5,6].

Vitamin D deficiency is a critical factor in the morbidity and mortality associated with neonatal sepsis. According to some theories, vitamin D promotes the synthesis of antimicrobial peptides in neutrophils, macrophages, and epithelial cells, thereby supporting the innate immune system. Research has shown that vitamin D levels in cord blood are associated with an increased risk of infections in newborns, and there is evidence linking vitamin D deficiency to neonatal sepsis in term infants [7].

Sepsis induces a range of pathophysiological changes, including excessive inflammation, immune system dysfunction, a hypermetabolic state, and damage to multiple organs, all of which are potential targets for vitamin D treatment. It has been shown that the VDR is present in immune cells and through the regulation of immune functions, 25-hydroxy vitamin D could help prevent sepsis. Additionally, vitamin D may boost the body's defenses against pathogens, modulate the adaptive immune response, guard against autoimmune diseases, and reduce graft rejection risks [4,8].

Numerous observational and laboratory studies have demonstrated vitamin D's anti-inflammatory properties, including its role in regulating the production of endogenous antimicrobial peptides. Maintaining sufficient vitamin D levels is therefore essential for bone health and may improve the body's response to infections. Vitamin D plays a significant role in the innate immune system by stimulating the production of antimicrobial peptides, such as LL-37, while modulating the inflammatory cascade triggered by lipopolysaccharide (LPS). Many neonates presenting with sepsis are found to be vitamin D deficient [9].

In neonates with EOS, vitamin D deficiency is linked to increased severity of sepsis and higher mortality rates. Sepsis is a major contributor to morbidity and mortality in neonates. Vitamin D is believed to support the production of antimicrobial peptides in immune cells, which could enhance the innate immune system's function [7].

Given the prevalence of vitamin D deficiency in neonates with sepsis, this study aims to investigate the association between vitamin D deficiency and sepsis in term neonates. The goal is to improve early diagnosis and management to reduce complications.

## Materials and methods

Study design

This case-control study was conducted in the Department of Pediatrics at Dr. D. Y. Patil Medical College, Hospital and Research Centre, Pune, from August 2022 to July 2024. Institutional ethics committee clearance (IESC/PGS2022/28) was secured before initiating the study to ensure adherence to ethical research standards. Additionally, informed written consent was obtained from the parents of all participating neonates.

Participation criteria

Inclusion criteria for the study encompassed term neonates up to 28 days of life, with cases defined as neonates presenting clinical and laboratory findings indicative of sepsis (Table 1) [10], while controls were term neonates without any clinical features of sepsis. Exclusion criteria included neonates with major systemic congenital anomalies and outborn neonates.

**Table 1:** Criteria of sepsis C-reactive protein (CRP)

Groups	Criteria
Group I: High probable sepsis	At least 3 sepsis-related clinical signs, CRP >10 mg/L, at least 2 other altered serum parameters in addition to CRP, blood culture; positive or negative
Group 2: Probable sepsis	Less than 3 sepsis-related clinical signs, CRP > 10 mg/L, at least 2 other altered serum parameters in addition to CRP Blood culture; negative
Group 3: Possible sepsis	Less than 3 sepsis-related clinical signs, CRP <10 mg/L, less than 2 other altered serum parameters, blood culture; negative
Group 4: No sepsis	No sepsis-related clinical signs, CRP <10 mg/L, no altered serum parameters, blood culture; negative

Sample size calculation

A total of 60 neonates were studied with 30 in the case group and 30 in the control group. The sample size was determined based on the mean and standard deviation of vitamin D levels in neonates with sepsis (16.0 ng/mL, SD 10.5) and without sepsis (29.07 ng/mL, SD 8.4). With a power of 95% and a significance level of 1%, and accounting for a 10% loss to follow-up, the sample size calculation was performed using WinPepi (version 11.65) software.

Data collection

A comprehensive history and clinical examination were performed for all subjects. The diagnosis of sepsis was made based on established criteria, and relevant maternal history, such as clinical chorioamnionitis and premature rupture of membranes, was documented to support the diagnosis. Blood samples were collected under aseptic conditions at the time when the sepsis was confirmed, with 0.5 mL of venous blood obtained from each subject for the evaluation of serum vitamin D levels. Vitamin D concentrations were measured using the chemiluminescent microparticle immunoassay (CMIA) method with the Architect Plus machine.

The vitamin D (25-hydroxy) levels were classified into three categories: sufficient (>20 ng/mL to 50 ng/mL), insufficient (12 ng/mL to 20 ng/mL), and deficient (<12 ng/mL) [11]. This classification was used to assess the vitamin D status of the subjects and its potential impact on their health.

Further evaluation and management

Based on the vitamin D levels and clinical findings, each subject underwent detailed evaluation and management following established protocols [12]. This approach ensured that all subjects received appropriate care based on their individual needs and vitamin D status.

Statistical analysis

Data were organized in Microsoft Excel (Microsoft® Corp., Redmond, WA) and analyzed using GraphPad Prism 10 software (GraphPad Software, San Diego, CA). The comparison of vitamin D levels between neonates with sepsis and those without sepsis was performed using Student's t-test, with significance set at a p-value of <0.05. The categorical variables were analyzed using the chi-square test. Additionally, z-scores were computed for frequency comparisons to assess differences in incidence rates. All statistical analyses were conducted with a 95% confidence interval.

## Results

**Figure 1:**
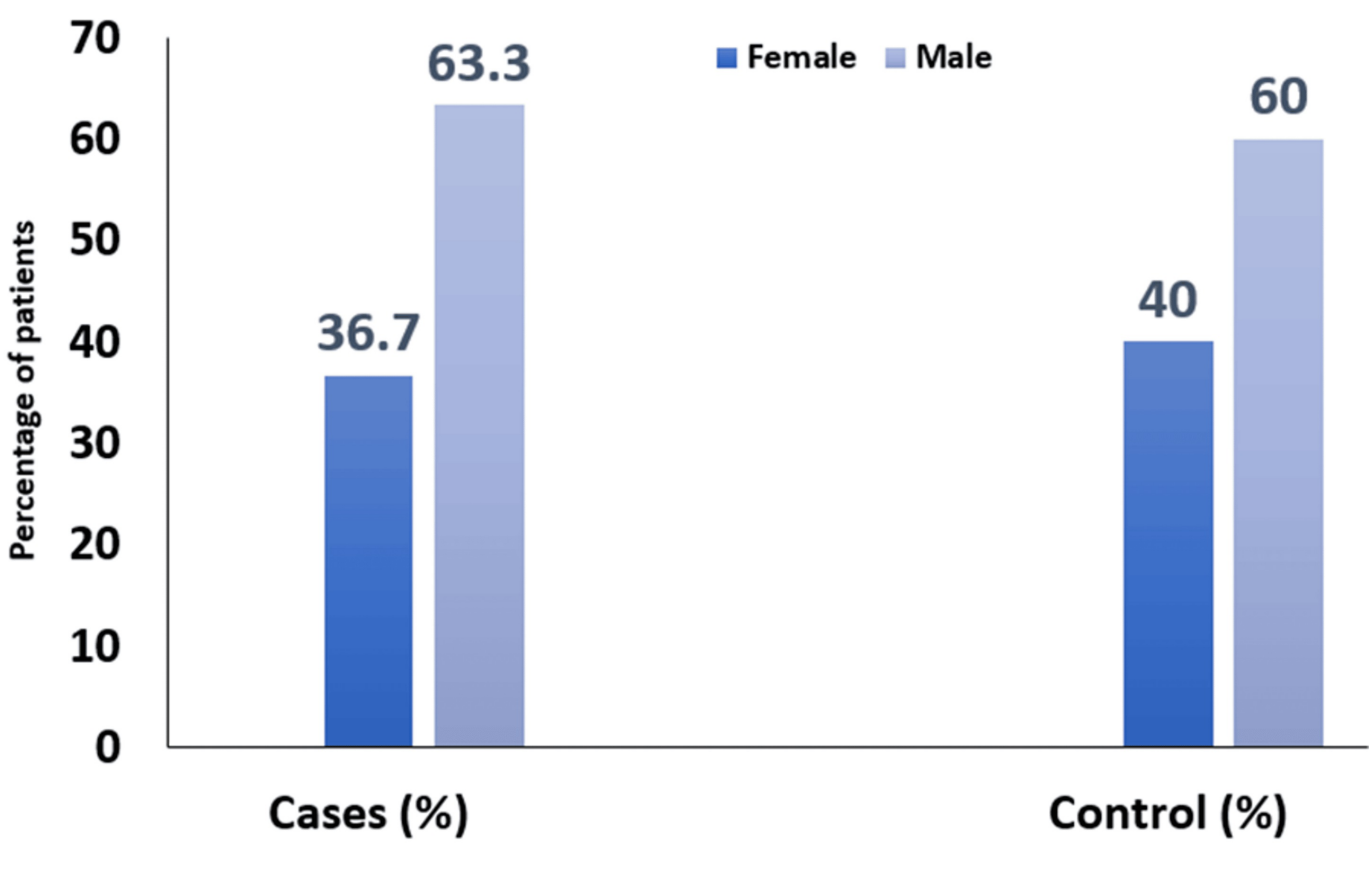
Distribution of population study based on gender into each group

Among the 30 neonates with sepsis, 11 (36.7%) were female and 19 (63.3%) were male. In the control group, which also consisted of 30 neonates without sepsis, 12 (40%) were female, and 18 (60%) were male. Overall, out of the total 60 neonates studied, 23 (38.3%) were female, and 37 (61.7%) were male (Figure 1).

**Figure 2:**
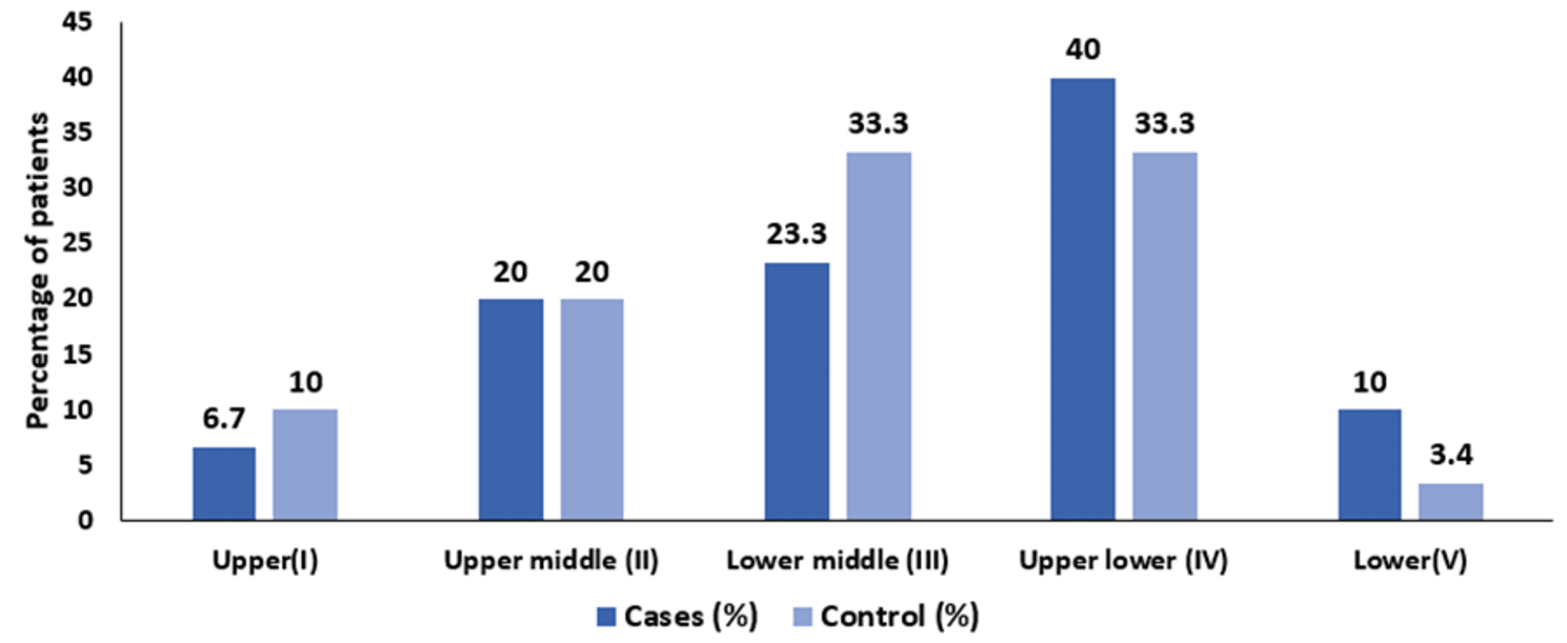
Distributions of subjects according to socioeconomic status

We analyzed the distribution of subjects according to socioeconomic status (Figure 2), as categorized using the Modified Kuppuswamy Scale. In the cases group (n=30), two (6.7%) were classified in the upper class, six (20%) in the upper middle class, seven (23.3%) in the lower middle class, 12 (40%) in the upper lower class, and three (10%) in the lower class. In the control group (n=30), three (10%) were in the upper class, six (20%) in the upper middle class, 10 (33.3%) in the lower middle class, 10 (33.3%) in the upper lower class, and one (3.4%) in the lower class. Overall, among the 60 subjects, five (8.3%) were in the upper class, 12 (20%) in the upper middle class, 17 (28.3%) in the lower middle class, 22 (36.7%) in the upper lower class, and four (6.7%) in the lower class.

**Figure 3:**
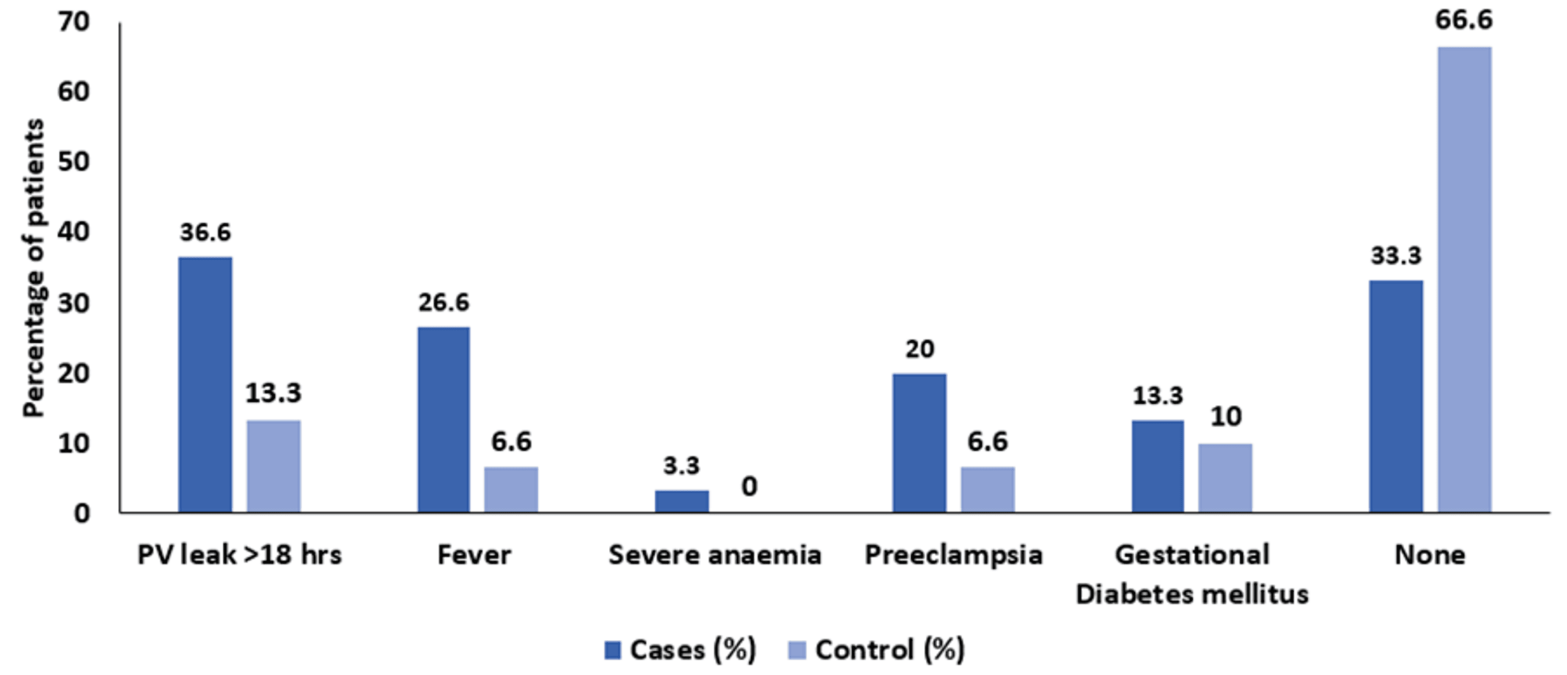
Distribution of subjects according to maternal risk factors PV = per vaginal

The impact of maternal risk factors on neonatal sepsis cannot be neglected. We therefore analyzed the distribution of subjects according to maternal risk factors. In the sepsis group, 11 mothers (36.6%) experienced a prolonged PV leak of more than 18 hours, compared to four mothers (13.3%) in the control group. Fever was reported in eight mothers (26.6%) of the sepsis group versus two mothers (6.6%) in the control group. Severe anemia was present in one mother (3.3%) in the sepsis group, while none were affected in the control group. Preeclampsia was observed in six mothers (20%) of the sepsis group and two mothers (6.6%) in the control group. Gestational diabetes mellitus was noted in four mothers (13.3%) of the sepsis group and three mothers (10%) of the control group. Additionally, 10 mothers (33.3%) in the sepsis group had no reported risk factors, while 20 mothers (66.6%) in the control group had no such risk factors (Figure 3).

The hemogram analysis of the study revealed significant differences between the case group and the control group across various parameters. The mean hemoglobin level in the cases was 13.81 g/dL (±2.76), notably lower than the control group's mean of 18.73 g/dL (±1.26), with a P value of less than 0.00001, indicating a highly significant difference. The total leukocyte count was also markedly lower in the cases group, averaging 6.11 (±3.33) x 10³/mm³ compared to 15.72 (±21.89) x 10³/mm³ in the control group, with a P value of less than 0.00001. Platelet counts showed a stark contrast as well, with the cases group having a mean of 94.59 (±63.92) x 10³/mm³, significantly lower than the control group's 279.23 (±72.50) x 10³/mm³, again with a P value of less than 0.00001. Neutrophil percentages were higher in the cases group at 54.00% (±23.27) compared to 40.17% (±6.59) in the control group, with a P value of 0.0009, suggesting an ongoing infection or inflammatory response. Conversely, lymphocyte percentages were lower in the cases group at 36.22% (±21.30) compared to 47.00% (±5.20) in the control group, with a P value of 0.0034, indicating a potential depletion or suppression of these immune cells. The absolute neutrophil count in the cases group was 3.39 (±2.47) x 10³/mm³, significantly lower than the control group's 6.213 (±1.21) x 10³/mm³, with a P value of less than 0.00001, further underscoring the compromised immune function in the cases group (Table 2).

**Table 2:** Hematological parameters in the study population T-test used to calculate p-value p-value <0.05, statistically significant

Hemogram constituents	Cases ( Mean ± SD)	Control ( Mean ± SD)	P-value
Haemoglobin (g/dL)	13.81 ± 2.76	18.73 ± 1.26	< 0.0001
Total leukocyte count (10^3^/mm^3^)	6.11 ± 3.33	15.72 ± 21.89	< 0.0001
Platelet count (x10^3^/mm^3^)	94.59 ± 63.92	279.23 ± 72.50	< 0.0001
Neutrophils (%)	54.00 ± 23.27	40.17 ± 6.59	0.0009
Lymphocytes (%)	36.22 ± 21.30	47 ± 5.2	0.0034
Absolute neutrophil count (10^3^/mm^3^)	3.39 ± 2.47	6.213 ± 1.21	< 0.0001

**Figure 4:**
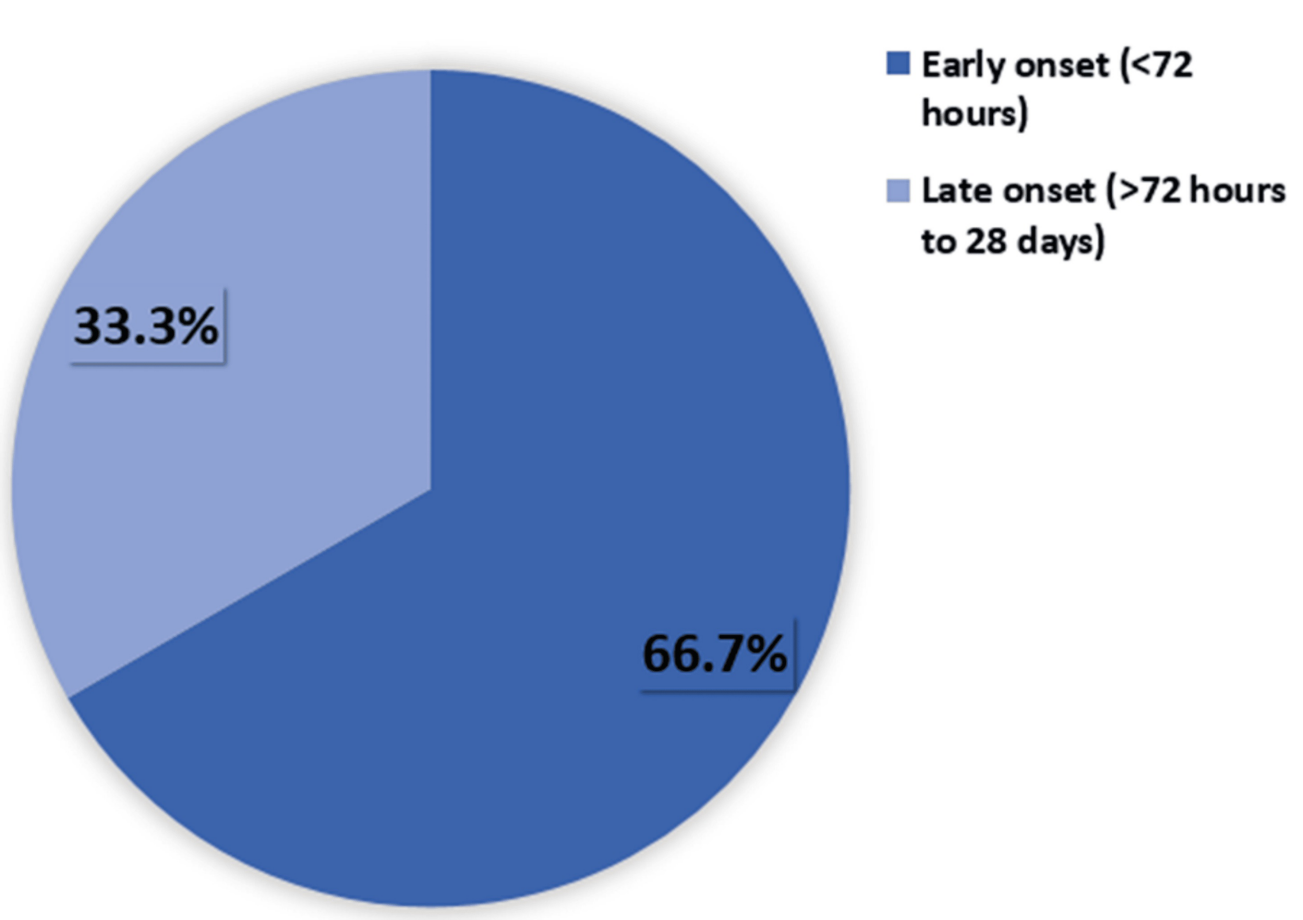
Day-wise clinical presentation of cases

We analyzed the timing of sepsis onset among 30 cases. The results revealed that the majority of sepsis cases were classified as EOS, occurring within the first 72 hours of life. Specifically, 20 out of the 30 cases (66.7%) presented with EOS. In contrast, the remaining 10 cases (33.3%) were categorized as LOS, which occurred between 72 hours and 28 days after birth (Figure 4).

**Figure 5:**
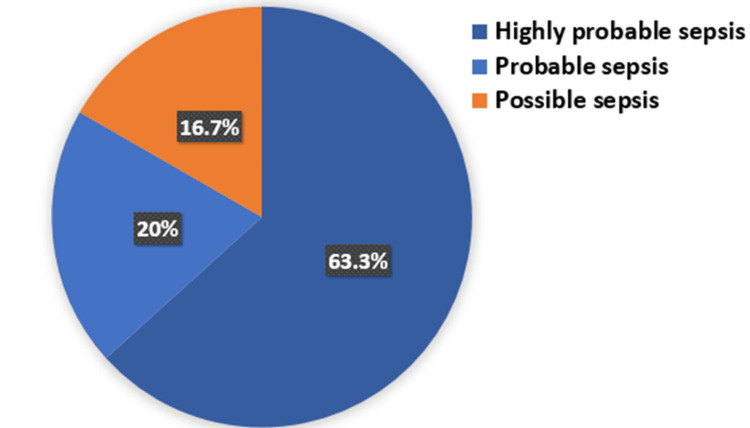
Distribution of cases according to clinical and laboratory criteria of sepsis

In our study, we categorized cases into three groups based on the likelihood of sepsis. Highly probable sepsis, probable sepsis, and possible sepsis. Out of the 30 cases analyzed, 19 cases (63.3%) were classified as highly probable sepsis, six cases (20%) as probable sepsis, and five cases (16.7%) as possible sepsis (Figure 5).

Among 30 patients in the case group, the blood culture results showed that, in 15 (50%) cases, the cultures were negative. Among the positive cultures, *Klebsiella pneumoniae* was identified in 11 cases (36.7%), *Enterococcus faecium* in two cases (6.7%), *Enterobacter cloacae* in one case (3.3%), and Methicillin-resistant *Staphylococcus aureus* in onnne case (3.3%) (Table 3).

**Table 3:** Distribution of cases based on the blood culture

Blood culture growth		Cases (n=30)	Percentage (%)
Present	Klebsiella pneumoniae	11	36.7
	Enterococcus faecium	2	6.7
	Enterobacter cloacae	1	3.3
	Methicillin-resistant *Staphylococcus aureus*	1	3.3
Absent		15	50
Total		30	100

Different clinical systems were affected in the case group patients. For the central nervous system (CNS), poor activity was observed in 12 cases (40%), hypotonia in 10 cases (33.3%), convulsions in eight cases (26.6%), and irritability in two cases (6.6%). In the respiratory system, tachypnoea was noted in 14 cases (46.6%), retractions in 11 cases (36.6%), nasal flaring in seven cases (23.3%), grunting in four cases (13.3%), and apnoea in two cases (6.6%). Cardiovascular system (CVS) involvement included tachycardia in 13 cases (43.3%), hypotension in 12 cases (40%), edema in six cases (20%), bradycardia in three cases (10%), and delayed capillary filling time in three cases (10%). Gastrointestinal (GIT) issues were abdominal distension in 10 cases (33.3%), vomiting in eight cases (26.6%), and not passing stool in three cases (10%). Hematological findings included bleeding with pallor in eight cases (26.6%) and purpura or ecchymosis in six cases (20%). Renal issues were oliguria in five cases (16.6%). Temperature instability was observed as hyperthermia in 12 cases (40%) and hypothermia in four cases (13.3%). Skin signs included mottling in seven cases (23.3%), icteric appearance in six cases (20%), and sclerema in four cases (13.3%) (Table 4).

**Table 4:** Clinical features-wise distribution of case group patients CNS = central nervous system; CVS = cardiovascular system; GIT = gastrointestinal tract

System Involved		Frequency (n)	Percentage (%)
CNS	Poor activity	12	40
	Hypotonia	10	33.3
	Convulsion	8	26.6
	Irritability	2	6.6
Respiratory	Tachypnoea	14	46.6
	Retractions	11	36.6
	Nasal flaring	7	23.3
	Grunting	4	13.3
	Apnoea	2	6.6
CVS	Tachycardia	13	43.3
	Hypotension	12	40
	Oedema	6	20
	Bradycardia	3	10
	Delayed capillary filling time	3	10
GIT	Abdominal distension	10	33.3
	Vomiting	8	26.6
	Not passing stool	3	10
Haematological	Bleeding with pallor	8	26.6
	Purpura or ecchymosis	6	20
Renal	Oliguria	5	16.6
Temperature Instability	Hyperthermia	12	40
	Hypothermia	4	13.3
Skin Signs	Mottling	7	23.3
	Icteric	6	20
	Sclerema	4	13.3

**Figure 6:**
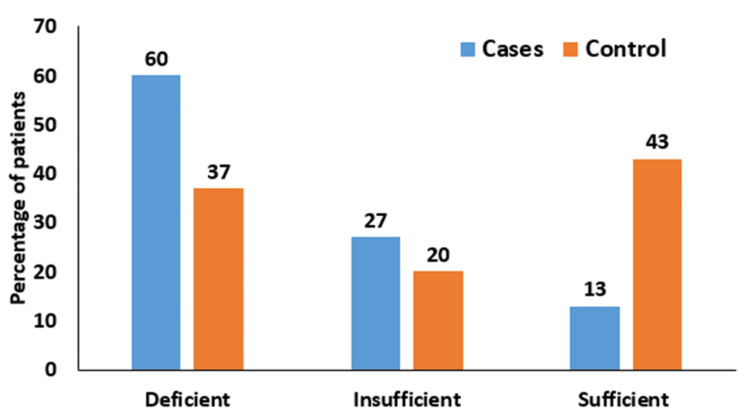
Distribution of vitamin D levels in subjects Deficient (<12 ng/dL); insufficient (12-20 ng/dL); sufficient (>20 ng/dL to 50 ng/mL)

Among the cases, 18 individuals (60%) were found to be vitamin D deficient (<12 ng/dL), eight individuals (26.7%) had insufficient levels (12-20 ng/dL), and four individuals (13.3%) had sufficient levels (>20 ng/dL to 50 ng/mL). In the control group, 11 individuals (36.7%) were vitamin D deficient, six individuals (20%) had insufficient levels, and 13 individuals (43.3%) had sufficient levels. Overall, combining both groups, 29 individuals (48.4%) were deficient, 14 individuals (23.3%) were insufficient, and 17 individuals (28.3%) had sufficient levels of vitamin D (Figure 6).

We further analyzed the onset and criteria of sepsis with vitamin D levels among participants (Table 5). For EOS, among those with vitamin D deficiency (n=18), eight cases (44.4%) were classified as highly probable, four cases (22.2%) as probable, and none (0.0%) as possible. Among those with insufficient vitamin D levels (n=8), three cases (37.5%) were highly probable, one case (12.5%) was probable, and two cases (25%) were possible. Among those with sufficient vitamin D levels (n=4), one case (25%) was highly probable, none (0.0%) was probable, and one case (25%) was possible.

**Table 5:** Distribution of cases based on the severity of sepsis and corresponding vitamin D levels

Onset of sepsis	Criteria of sepsis	Vitamin-D Deficient n (%)	Vitamin-D Insufficient n (%)	Vitamin-D Sufficient n (%)
Early onset sepsis	Highly probable	8 (44.4)	3 (37.5)	1 (25)
	Probable	4 (22.2)	1 (12.5)	0 (0.0)
	Possible	0 (0.0)	2 (25)	1 (25)
Late-onset sepsis	Highly probable	4 (22.2)	2 (25)	1 (25)
	Probable	1 (5.6)	0 (0.0)	0 (0.0)
	Possible	1 (5.6)	0 (0.0)	1 (25)

For LOS, among those with vitamin D deficiency, four cases (22.2%) were classified as highly probable, one case (5.6%) as probable, and one case (5.6%) as possible. Among those with insufficient vitamin D levels, two cases (25%) were highly probable, none (0.0%) were probable, and none (0.0%) were possible. Among those with sufficient vitamin D levels, one case (25%) was highly probable, none (0.0%) was probable, and one case (25%) was possible.

## Discussion

Neonatal sepsis is the cause of major morbidity and mortality in infants. Neonatal sepsis is categorized as EOS (<72 hours) and LOS (>72 hours) neonatal sepsis [1]. Vitamin D deficiency is a key factor causing mortality and morbidity in neonatal sepsis. Vitamin D promotes the synthesis of antimicrobial peptides in neutrophils, macrophages, and epithelial cells, which could lead to the innate immune system's successful functioning [6,7].

Our study showed that, in the case group, 11 (36.6%) had PV leak > 18 hours, eight (26.6%) mothers had a history of fever, one (3.3%) had severe anemia, six (20%) had pre-eclampsia, four (13.3%) mothers had gestational diabetes mellitus, and 10 (33.3%) had no risk factors, while in control group, four (13.3) had PV leak > 18 hours, two (6.6%) mothers had history of fever, two (6.6%) had pre-eclampsia, three (10%) had gestational diabetes mellitus, and 20 (66.6%) had no risk factors. In a study by Gupta et al. in 2020, premature rupture of membrane (PROM) was the most common risk factor followed by fever, anemia, pre-eclampsia, and hypertension [11]. In the study conducted by Salama et al. in 2023, the most influential predictor of neonatal sepsis was PROMs [13]. Similarly, in Adatara et al., PROM had a significant association with the risk of neonatal sepsis [14].

In this study, we showed 13 (43.3%) cases were delivered by vaginal delivery and 17 (56.7%) cases by lower segment cesarean section (LSCS), 21 (70%) controls were delivered by vaginal delivery, and nine (30%) by LSCS. Salama et al. showed that LSCS was highly associated with neonatal sepsis and neonates born via LSCS had two times more risk of having sepsis in comparison to those born via vaginal delivery [13].

In our study, among the 30 cases, CNS manifestations observed were poor activity (12, 40%), hypotonia (10, 33.3%), convulsions (8, 26.6%), and irritability (2, 6.6%). Common respiratory clinical features observed as tachypnoea (14 cases, 46.6%), retractions (11 cases, 36.6%), nasal flaring (7 cases, 23.3%), grunting (4 cases, 13.3%), and apnoea (2 cases, 6.6%). The common cardiovascular system manifestations observed were tachycardia (13 cases, 43.3%), hypotension (12 cases, 40%), edema (6 cases, 20%), bradycardia (3 cases, 10%), and delayed capillary filling time (3 cases, 10%). Gastrointestinal symptoms included abdominal distension in 10 (33.3%), vomiting in eight (26.6%), and not passing stool in three (10%) cases. Hematological manifestations observed were bleeding with pallor (8, 26.6%) and purpura or ecchymosis (6, 20%) cases. Renal involvement was observed as oliguria in five (16.6%) cases. Hyperthermia in 12 (40%) and hypothermia in four (13.3%) cases presented as temperature instability. Skin mottling in seven (23.3%), jaundice in six (20%), and sclerema in four (13.3%) cases were observed as skin manifestations. Gupta et al. showed that the leading clinical manifestation was respiratory distress, followed by apnoea and hypothermia [11].

In this study, EOS was present in 20 cases (66.7%), and LOS was present in 10 cases (33.3%) with LOS. In the study by Ozdemir et al., among 51 newborns diagnosed with sepsis, the majority (76.5%) had EOS, while 23.5% had LOS [15]. In another study by Roble et al., out of the total 163 neonates diagnosed with sepsis, the majority (131, 80.4%) were identified as having EOS, while 32 (19.6%) were diagnosed with LOS [16].

Among 30 cases, 15 blood cultures were positive showing bacterial growth of *K. pneumoniae* in 11 (36.7%) cases, *Enterococcus faecium* in two (6.7%) cases, *Enterobacter cloacae* was identified in one (3.3%) case, and MRSA in one (3.3%) case. In Agrawal et al.'s study, the predominant organism isolated from blood cultures was *E. coli *(57 cases, 32.5%), followed by *Klebsiella *(42 cases, 24%), *Pseudomonas *(23 cases, 13.14%), Group B *Streptococcus *(17 cases, 9.7%), *S. aureus *(11 cases, 6.2%), and coagulase-negative *Staphylococcus *(8 cases, 4.5%) [17]. In the study conducted by Singh et al., blood cultures of neonates with sepsis were Coagulase-negative *Staphylococcus aureus* (21.42%), *Klebsiella *(12.86%), and *Acinetobacter *(10%) [7]. In the study conducted by Olorukooba et al., 37.6% had positive blood cultures with *E. coli* (31%), *S. aureus* (18%), and *K. pneumoniae* (11%) [18]. In another study by Mohamed et al., out of 45 cases, 26 (57.8%) had a positive blood culture, while 19 (42.2%) showed no growth in blood culture. Among the positive blood culture cases, *Klebsiella* was found in seven (26.9%), coagulase-negative staphylococci in 17 (65.4%), *E. coli* in one (3.8%), and *Acinetobacter *in one (3.8%) [19].

Among 30 cases, 18 (60%) were diagnosed with vitamin D deficiency, eight (26.7%) with vitamin D insufficiency, and four (13.3%) with sufficient vitamin D levels. In contrast, among the control group, 11 (36.7%) were diagnosed with vitamin D deficiency, six (20%) with vitamin D insufficiency, and 13 (43.3%) with sufficient vitamin D levels. A study done by Singh et al. showed that neonatal vitamin D was significantly lower in terms of EOS in comparison to those without sepsis (p=0.001) [7]. In the study by Perveen et al., the mean vitamin D level was 21.47 ng/mL ± 7.16 ng/mL, which showed a significant difference compared to the control group (18.14 ng/mL ± 7.07 ng/mL) with a p-value of 0.035. Among the 82 cases analyzed, 17% had sufficient vitamin D levels (> 30 ng/mL). The septic group exhibited 88% of cases with vitamin D deficiency, whereas the non-septic group had 78%, which was a statistically significant difference [20]. In the study conducted by Ozdemir et al., it was found that the prevalence of vitamin D deficiency was significantly higher in the sepsis group (60.8%, n = 31) compared to the control group (53.6%, n=30; p=0.00). Additionally, the sepsis group exhibited significantly lower levels of vitamin D (11 ± 5.5 ng/mL) compared to the control group (13.8 ± 10.6 ng/mL; p=0.012) [15].

We further showed that in EOS, out of 20 cases, 12 were highly probable sepsis. Of these, eight (44.4%) were diagnosed with deficient vitamin D, three (37.5%) with insufficient vitamin D, and one (25%) with sufficient vitamin D. Among the five cases of probable sepsis, four (22.2%) had deficient vitamin D, and one (12.5%) had insufficient vitamin D. Out of three cases of possible sepsis, two (25%) had insufficient vitamin D, and one (25%) had sufficient vitamin D. In LOS, out of 10 cases, seven were highly probable sepsis. Of these, four (22.2%) had deficient vitamin D, two (25%) had insufficient vitamin D, and one (25%) had sufficient vitamin D. There was one (5.6%) case of probable sepsis with deficient vitamin D. Among two cases of possible sepsis, one (5.6%) had deficient vitamin D, and one (25%) had sufficient vitamin D. The difference was statistically insignificant (p=0.910). In Singh et al.'s study, among 70 cases of EOS, 62.1% of highly probable sepsis cases had vitamin D deficiency, 18.6% had vitamin D insufficiency, and 28.6% had sufficient vitamin D levels. For probable sepsis cases, 13.8% were found to have vitamin D deficiency, 44.4% with vitamin D insufficiency, and 21.4% with sufficient vitamin D levels. Among possible sepsis cases, 24.1% had vitamin D deficiency, 37% had vitamin D insufficiency, and 50% had sufficient vitamin D levels. The severity of vitamin D deficiency correlated with the severity of sepsis [7]. Ozdemir et al.'s study revealed that newborns with EOS had a mean vitamin D level of 10.4 ± 5.7 ng/mL, while those with LOS had a mean level of 12.8 ± 4.3 ng/mL [15].

This study adds valuable insights to the field in determining the importance of vitamin D supplementation in high-risk neonates. However, we acknowledge several limitations of this study. The small sample size is the major limitation, which may limit the application of the study findings to a broader population. Additionally, the study has not accounted for potential confounding factors such as maternal vitamin D status, environmental influences, maternal systemic diseases, and congenital diseases that could affect neonatal vitamin D levels. The study was conducted in a single center, which may limit the applicability of the results to different settings.

## Conclusions

Our study concluded that serum vitamin D levels were significantly lower in septic neonates compared to healthy controls, suggesting a potential role of vitamin D deficiency in the pathogenesis of sepsis. We observed that EOS was more prevalent than LOS, highlighting the need for early vitamin D supplementation in high-risk neonates to prevent the severity and complications associated with sepsis. Additionally, normal neonates exhibited low vitamin D levels, indicating that universal vitamin D supplementation at birth, coupled with maternal vitamin D supplementation, may be beneficial for all neonates to address this deficiency and improve overall neonatal health.
